# Adsorption Features of Various Inorganic Materials for the Drug Removal from Water and Synthetic Urine Medium: A Multi-Technique Time-Resolved In Situ Investigation

**DOI:** 10.3390/ma14206196

**Published:** 2021-10-19

**Authors:** Enrico Boccaleri, Cristina Marzetti, Giorgio Celoria, Claudio Cassino, Geo Paul, Ivana Miletto, Enrica Gianotti

**Affiliations:** UPO4Sustainability Centre, Dipartimento di Scienze ed Innovazione Tecnologica (DiSIT), Università del Piemonte Orientale, Viale T. Michel 11, 15121 Alessandria, Italy; cristina.marzetti@yahoo.it (C.M.); giorgio.celoria@uniupo.it (G.C.); claudio.cassino@uniupo.it (C.C.); enrica.gianotti@uniupo.it (E.G.)

**Keywords:** pharmaceutical active compounds, adsorption, inorganic adsorbents, ibuprofen, X-ray powder diffraction, solid-state NMR, Raman spectroscopy, time-resolved in situ studies, water remediation

## Abstract

Pharmaceutical active compounds, including hundreds of different substances, are counted among the emerging contaminants in waterbodies, whose presence raises a growing concern for the ecosystem. Drugs are metabolized and excreted mainly through urine as an unchanged active ingredient or in the form of metabolites. These emerging contaminants are not effectively removed with the technologies currently in use, making them a relevant environmental problem. This study proposes the treatment of urine and water at the source that can allow an easier removal of dissolved drugs and metabolites. The treatment of synthetic urine, with dissolved ibuprofen as a model compound, by adsorption, using various classes of inorganic materials, such as clays, hierarchical zeolites and ordered mesoporous silica (MCM-41), is presented. A multi-technique approach involving X-ray powder diffraction, solid-state NMR, UV-Vis and Raman spectroscopies was employed to investigate the adsorption process in inorganic adsorbents. Moreover, the uptake, the ensuing competition, the efficiency and selectivity as well as the packing of the model compound in ordered mesoporous silica during the incipient wetness impregnation process were all thoroughly monitored by a novel approach, involving combined complementary time-resolved in situ ^1^H and ^13^C MAS NMR spectroscopy as well as X-ray powder diffraction.

## 1. Introduction

Water is a vital resource for all living organisms [1,2] and its availability, use and quality are harmed by its unequal distribution, climate change, population growth, increasing consumption, and waste; however, a crucial aspect regarding both water quality and environmental impact is connected to pollution related to wastewater streams [3]. Besides the so-called priority pollutants (PPs), related to significant risk for the aquatic environment and human health, a multitude of compounds of anthropogenic origin, highlighting a global problem, is to be tackled [4,5,6]. Among them, emerging contaminants (EC) can potentially cause adverse effects on the ecosystem and human health [7]. Besides particles as microplastics, a plethora of water-soluble emerging contaminants should be considered, including pesticides, surfactants, artificial sweeteners, additives, drugs and active pharmaceutical ingredients (APIs), compounds contained in products for care and personal hygiene (pharmaceuticals and personal care products, PPCPs) and, in particular, endocrine-disrupting compounds. The expansion of the pharmaceutical industry has promoted the release and spread of pharmaceutical active compounds (PhACs) in the environment to such an extent that today, they are counted among the emerging contaminants whose presence raises a growing concern to the ecosystem [3,8,9,10,11].

The PhACs category includes thousands of different compounds: in addition to drugs employed for human use, it also includes those for veterinary use, illegal drugs and metabolites of these compounds. The active pharmaceutical ingredients are specifically engineered to be easily absorbed by organisms, to be bioactive even at low concentrations, to have good stability and a prolonged half-life [12]. After administration, drugs are metabolized and excreted mainly through the urine (64% of PhACs are eliminated by the kidneys) and feces, either as unmodified compounds or in the form of metabolites [13].

Conventional wastewater treatment plants (WWTPs) are responsible for the reclamation of wastewater from a wide selection of pollutants, but they are not specifically designed for the removal of so-called emerging contaminants: none of the treatments currently in use, conventional or advanced, nor their synergistic application, are able to guarantee a complete removal of ECs from wastewater [7]. Part of this lack of efficiency against ECs is due to the mixing and dilution of highly heterogeneous streams, i.e., outflows of the hospital sector, which make a strong contribution to the load of pharmaceutical compounds present in civil wastewater [14,15,16,17], together with rain, washing, rinsing, flushing, etc.

For the wide therapeutical address and the limited presence of side effects, nonsteroidal anti-inflammatories (NSAIDs) are a therapeutic category whose incidence in the environment is much studied; among them, ibuprofen (Ibu-Na), diclofenac (DIC), paracetamol, ketoprofen (KET) and naproxen (NAP) have been observed in high concentration both in tributaries and effluents of WWTPs. The overall results of more than 50 evaluation assays conducted in 20 different countries reported the presence of diclofenac and ibuprofen in more than 90% of the samples analyzed [18]. In addition, levels of tens of ng/g of Ibu-Na and DIC have been observed in some marine species, such as in the mussels of Italian Adriatic coasts [19] and in the Portuguese Atlantic coasts [20].

To date, adsorption is one of the most promising technologies for the removal of active pharmaceutical ingredients from water, but one of the critical issues is the low concentration of these molecules at the sewage plant level. Due to the relevance of this anthropogenic problem, the present study was carried out, using ibuprofen as test API and inorganic matrixes with different morphological and structural features as adsorbents. The scope of this research is grounded on the possibility to treat water and urine directly at the excretion source (i.e., hospitals or nursing homes) in order to allow a more efficient removal of drugs, as the concentration is higher, and the treatment can be done in local controlled environments.

This study aims at identifying potential inorganic adsorbents for the removal of active pharmaceutical ingredients at low concentration as typically found in water and urine, besides the plethora of other chemical species present in the latter complex medium. A multi-technique approach involving X-ray powder diffraction, solid-state NMR, UV-Vis and Raman spectroscopies was employed to investigate the non-covalent interactions responsible for adsorption process. In order to follow the fundamental mechanistic steps of encapsulation progression and complex molecular adsorption during the incipient wetness impregnation process, a time-resolved in situ study is essential [21,22]. Therefore, the local packing behavior of ibuprofen on the mesopores of MCM-41 was monitored, using a combined complementary time-resolved in situ MAS NMR spectroscopy and powder XRD.

## 2. Materials and Methods

(±)-2-(4-Isobutylphenyl)propanoic acid or α-methyl-4-(isobutyl)phenylacetic acid, more commonly known as ibuprofen in acid form, was acquired from Sigma-Aldrich (St. Louis, MO, USA) and used as is. The sodium salt of ibuprofen (Ibu-Na) in granulated formulation (with traces of NaHCO_3_) was a gift from a pharmaceutical company. Sodium chloride, magnesium chloride, calcium chloride, potassium chloride, sodium phosphate, sodium sulfate and sodium citrate tribasic dihydrate were supplied by Sigma-Aldrich (Steinheim, Germany). Urea was supplied by Fluka (Buchs, Switzerland). Physiological saline solution was purchased from Eurospital S.p.A (Trieste, Italy).

Synthetic urine employed during experiments was prepared according to Table S1 [23,24,25]. To determine the expected amount of Ibu-Na in urine, the following considerations were taken. The maximum prescribable daily dose is 1200 mg per day, and 70–90% of the administered dose, both orally and intravenously, is excreted in the urine in the first 24 h following the administration in the form of Ibu-Na and its catabolites, the latter representing the predominant fraction. Therefore, only 1–5% of the active substance is recovered in the urine [26,27]. These considerations were employed in the formula below:

$$\left[C\right]=\frac{DDD\times F\times 1000}{V_{urine}}$$

where [*C*] is the initial concentration in mg/L of drug in the urine; *DDD* (daily defined dose) is the daily dose of the administered active ingredient (mg/day); *F* (%) is the unchanged excreted drug fraction through the urine; *V_urine_* is the average daily urine volume (1500 mL); and 1000 is the conversion factor from mL to L. Table S2 summarizes the parameters and the concentrations of active substance used. Citrate was selected as the interfering reference metabolite, as reported in [25].

Fresh urine was prepared each day in order to avoid possible urea hydrolysis and compositional changes due to aging and storage conditions. The contained salts were dissolved in 1 L of deionized H_2_O, and the flask was wrapped with aluminum foil to protect it from light and sonicated for 20 min. The pH was measured with a Hanna HI98108 pH meter (Smithfield, RI, USA) and adjusted with concentrated solutions of NaOH or HCl.

Inorganic adsorbents: Cloisite-Na^+®^ and Cloisite-Ca^++®^ are two natural bentonites marketed as fillers for several applications (i.e., as polymer additives). They were purchased from Southern Clay Products (Gonzales, TX, USA). The typical particle size is between 2 μm and 13 μm; the interlamellar distance is 12 Å and 15 Å, respectively; and the cation exchange capacities (CEC) provided by the manufacturer are 92.6 meq/100g and 96 meq/100g, respectively. From the literature, it is known that the external specific surface area (SSA) is 30–32 m^2^/g [28], but following exfoliation, it reaches values in the range of 725–800 m^2^/g [29,30,31]. Molecular sieves (before and after activation) in pellet form (3.2 mm) with a pore diameter of 4 Å were purchased from Sigma-Aldrich (Steinheim, Germany). It was activated by thermally treating the material at 150 °C for 24 h.

Microporous NH_4_-ZSM-5 zeolite (SiO_2_/Al_2_O_3_ = 80) was provided by Zeolyst International (Kansas City, KS, USA). To obtain a hierarchical porous architecture (Hier ZSM-5), the microporous NH_4_-ZSM-5 was desilicated following the procedure of Erigoni et al. [32]. According to the literature, the textural features show a S_BET_ of 500 m^2^/g, S_meso_ of 215 m^2^/g, V_pore_tot_ equal to 0.81 cm^3^/g, V_meso_ cm^3^/g of 0.7, and pore diameter_avg_ of 60 Å.

Calcined MCM-41 was purchased from Sigma-Aldrich (Steinheim, Germany); the average pore size provided by the manufacturer is 21–27 Å, the specific surface area (SSA) is ca. 1000 m^2^/g, and the pore volume is 0.98 cm^3^/g.

MCM-41 nanoparticles (MCM-41 NPs) were synthesized according to the procedure by Miletto et al. [33]. In a typical synthesis, 1 g of CTAB (hexadecyltrimethylammonium bromide) and 3.5 mL of 2M NaOH were added to 480 mL of deionized water in a three-necked flask. The mixture was brought to 80 °C in an oil bath under magnetic stirring, and then 5 mL of TEOS (tetraethyl orthosilicate) was added drop by drop. The reaction mixture was further stirred for 2 h, after which the solution was cooled in an ice bath. The precipitate obtained was separated by filtration and washed with deionized water (1 L) and with ethanol (200 mL). The obtained material was dried at 60 °C overnight in an oven and calcined at 560 °C in a tubular oven (Lenton, U.K.) with a temperature ramp of 2 °C per minute in N_2_ flow; this temperature was maintained for 8 h in O_2_ flow. The textural characteristics of spherical MCM-41 NPs were previously reported [34]. The average pore size is 33 Å, the specific surface area (SSA) is 1097 m^2^/g and the pore volume is 1.36 cm^3^/g.

Merck grade silica gel was purchased from Sigma-Aldrich (Steinheim, Germany) (grade 60, 70–230 mesh, 60 Å, a SSA of 470–530 m^2^/ g, and a volume of 0.7–0.85 cm^3^/g). Fumed silica was provided by Sigma-Aldrich (Steinheim, Germany) as amorphous SiO_2_ with low a density and high surface area (395 m^2^/g).

The Ibu-Na stock solution and the sorbents in a ratio of 5 g/L were dosed in 50 mL polypropylene tube, subjected to vigorous stirring for 30 s with the aid of a vortex mixer to obtain a good dispersion, and placed in a horizontal position in an incubator with orbital stirring, Stuart SI 50 Bibby Scientific™ (Fisher Scientific, Rodano, Milano, Italy), at a speed set at 180 oscillations per minute and at a temperature of 20 °C. After 2 h of contact, the samples were centrifuged at 8000 rpm for 10 min. The supernatant was analyzed by UV-visible spectrophotometry (Lambda900 Perkin Elmer, Waltham, MA, USA), using the Lambert–Beer law.

A concentration calibration was performed, using the Ibu-Na stock solution and the following dilutions; 1:2, 1:5, 1:10, 1:50 and 1:100. For each of the calibration equations, obtained with a least squares linear regression, the resulting R^2^ was greater than 0.998. For all samples, an aliquot of 3 mL and a quartz cuvette with an optical path of 1 cm were used.

As the LOD (limit of detection of the technique), the concentration corresponding to the dilution 1:50 was considered: in each of the cases, the signal of further dilutions was not statistically different from that of the solvent. The percentage adsorption of each material can be calculated with the following formula:

$$Ads\%=\frac{C_{f}\left[mol/L\right]}{C_{i}\left[mol/L\right]}\times 100\;\;\;\;\;\;\;\;\;$$

where *C_f_* and *C_i_* are the final and initial concentrations, respectively. The loading of therapeutic agent for each sample was obtained by applying the following equation:

$$loading\;\left[\frac{mg}{g}\right]=\frac{\left(C_{i}-C_{f}\right)\times V\times PM}{M}\times 1000\;$$

where *C_i_* is the concentration of the drug stock solution [mol L^−1^]; *C_f_* is the molar concentration of the supernatant at the end of the adsorption experiment [mol L^−1^]; *V* is the volume of solution used in the test [L]; *PM* is the molecular weight of the drug [g mol^−1^]; *M* is the mass of adsorbent used [g]; and 1000 is the conversion factor from g to mg of adsorbed active substance

Sample preparation for in situ experiments: A physical mixture was prepared by mixing MCM-41 and Ibu-Na in equal amounts (weight-to-weight proportions) and grinding on an agate mortar with a pestle. The prepared mixture was used for all the experiments. MCM-41 was impregnated with Ibu-Na by the incipient wetness procedure. During the in situ incipient wetness procedure for solid-sate NMR experiments, the physical mixture was packed into a Kel-F insert and hydrated with a minimum amount of deuterated water (Sigma-Aldrich, 99.9%), using a water to solid ratio (w/s) of around 1.3, and stirred for one minute [21]. After that, the Kel-F insert was rapidly sealed with a plug and a screw to suppress the escape of fluids, and fitted into a 4 mm rotor, closed with a Kel-F cap and analyzed (Scheme S1). The sample temperature (25 °C) was set, using a temperature control unit that was previously calibrated, using lead nitrate. Data acquisition was started within 30 min of the initial contact of physical mixture with deuterated water. The time intervals between patterns were varied, and selected patterns were plotted for clarity.

Similarly, for the in situ X-ray diffraction measurements, the physical mixture of MCM-41 and Ibu-Na was mixed with a minimum amount of milliQ water to prepare a paste; this was placed on a powder XRD airtight specimen holder, immediately covered with a dome to minimize its interaction with the external atmosphere, and analyzed (Scheme S1).

The mechanoloaded sample was prepared by hand grinding a physical mixture of MCM-41 and Ibu-Na, in the 2 to 1 weight-to-weight proportion in an agate mortar with a pestle for 30 min. The powder XRD measurements on the sample showed no residual peaks associated to crystalline Ibu-Na.

The synthetic urine-based sample for Raman spectral measurements was prepared according to the following procedure. The Ibu-Na stock solution and MCM-41 were dosed in a ratio of 5 g/L in a 50 mL polypropylene tube, stirred for 30 s and placed in an incubator with orbital stirring at a temperature of 20 °C. After 2 h of contact, the sample was centrifuged at 8000 rpm for 10 min; the solid part was collected and dried overnight in an oven at a temperature of 50 °C. The sample was named MCM-41\Ibu-Na_U. Its Raman spectrum was measured without any further treatment. 

The Raman spectra of the samples were acquired on a microRaman LabRam-HR Evolution instrument, Horiba Jobin Yvon Scientific (Paris, France), equipped with a Peltier effect-cooled CCD chamber, using a 532 nm Nd:YAG Laser with 80mW power as an excitation source. The setting of the microspectrometer was as follows: 50X lens, slit aperture 200 μm, and lattice 1800 lines/mm^2^. The acquisition time for each spectral window and the number of accumulations were optimized for each individual material, collected at room temperature. A silicon wafer with Raman signal at 520.7 cm^−1^ was used to calibrate the instrument daily. The spectra were acquired with labspec6 software (Horiba) and processed with OPUS-Spectroscopic software (Bruker Optik GmbH, Ettlingen, Germany) by means of cosmic ray removal operations, when necessary, smoothing, and baseline subtraction by polynomial reference curve generation. Unless otherwise specified, the Raman spectra were normalized by applying the software’s Min–Max method.

Solid-state NMR spectra were acquired on a Bruker Avance III 500 spectrometer (Faellanden, Switzerland) using a wide bore 11.75 Tesla magnet with operational frequencies for ^1^H and ^13^C of 500.13 and 125.76 MHz, respectively. A 4 mm triple resonance probe, in double resonance mode, with magic angle spinning (MAS) was employed in all the in situ experiments. The samples were hydrated in a Kel-F insert that was fitted into a zirconia rotor and spun at a MAS rate of 12 kHz, using a Bruker MAS III pneumatic unit. ^1^H MAS NMR spectra were collected with an excitation pulse of 100 kHz. For the ^13^C cross-polarization (CP) magic angle spinning experiments, the proton radio frequencies (RF) of 55 and 28 kHz were used for initial excitation and decoupling, respectively. During the CP period, the ^1^H RF field was ramped using 100 increments, whereas the ^13^C RF field was maintained at a constant level. During the acquisition, proton decoupling was applied (spinal-64 decoupling scheme). A moderate ramped radio frequency field of 55 kHz was used for spin locking, while the carbon RF field was matched to obtain optimal signal (40 kHz). The delays, d1, between accumulations were 3 and 2 s for ^13^C and ^1^H, respectively. The chemical shifts are reported using δ scale and are externally referenced to TMS at 0 ppm.

Additional ^1^H and ^13^C NMR measurements were done in the liquid state. The spectra were recorded using Bruker Avance Neo spectrometer (Faellanden, Switzerland) equipped with an 11.74 T magnet (Larmor frequency of 500.38 MHz for ^1^H) and a 5 mm double resonance Bruker Z-gradient Smart Probe and Bruker BSVT unit for temperature control. The ^1^H and ^13^C NMR spectra were recorded in deuterium oxide (D_2_O) using RF pulses of 22 and 28 kHz, 16 and 1024 scans, and a recycle delay of 5 and 3.5 s, respectively, at 300 K after waiting 10 min for sample temperature stabilization. The chemical shifts are reported using δ scale and are externally referenced to TMS at 0.0 ppm.

The powder XRD analyses were performed with a Bruker D8 Advance diffractometer (Karlsruhe, Germany) with Bragg–Brentano geometry, with Cu anode (radiation 1.5418 Å), equipped with a Ni filter and operating at 40 kV and 40 mA. For non-mesoporous samples, the 2θ interval explored was 5–60°, with a time per step of 0.5 s and automatic synchronization of the anti-scatter knife; the illumination area of the sample was fixed at 17 mm^2^. For MCM-41 and MCM-41 NPs, the 2θ range measured was 1- 10°, the height of the knife was set to 0.4 mm, the area of illuminated sample and time per step were, respectively, 17 mm^2^ and 0.5 s.

## 3. Results and Discussion

### 3.1. Ibu-Na Adsorption from Physiological Saline Solution

Nine different classes of inorganic adsorbents, tested for the removal of Ibu-Na from the physiological saline solution, were selected considering their capacity for binding drugs based on either ionic or non-covalent interactions. In Table 1, a comparative evaluation of the maximum adsorption capacity of these adsorbents toward Ibu-Na from physiological saline solution is reported.

The ordered mesoporous silicas (MCM-41 and MCM-41 NPs) exhibited the best adsorption capacity followed by hierarchical ZSM-5, amorphous silica and clays. To further elucidate the physisorption mechanism and local packing of Ibu-Na into the surface of the ordered mesoporous silica, time-resolved in situ MAS NMR spectroscopy and X-ray powder diffraction studies were coupled.

### 3.2. Time-Resolved In Situ ^13^C and ^1^H MAS NMR Spectroscopy

To investigate the dynamics of the adsorption process of an active pharmaceutical ingredient on ordered mesoporous silica (MCM-41), a time-resolved study was performed. The role of water in the interaction between active pharmaceutical ingredients and MCM-41 surface was unclear from previous studies. It is assumed that the strong affinity between water molecule and surface silanols may influence the adsorption of active pharmaceutical compounds.

Highly resolved ^13^C NMR spectra of Ibuprofen can be obtained by either MAS or CPMAS techniques and are extremely useful for determining the physical state of organic molecules adsorbed on mesoporous materials. The ^13^C CPMAS is based on the transfer of magnetization between ^1^H and ^13^C following heteronuclear dipolar coupling through space and is, therefore, sensitive only to rigid molecules. On the other hand, the ^13^C MAS NMR technique is able to detect both rigid and very mobile species, but using a short relaxation time interval between accumulations, only the latter can be selectively detected. The two techniques are, therefore, complementary and are used in synergy in this case, together with the acquisition of ^1^H MAS NMR spectra, to obtain important information on the state of Ibu-Na physisorbed on MCM-41.

**Figure 1:**
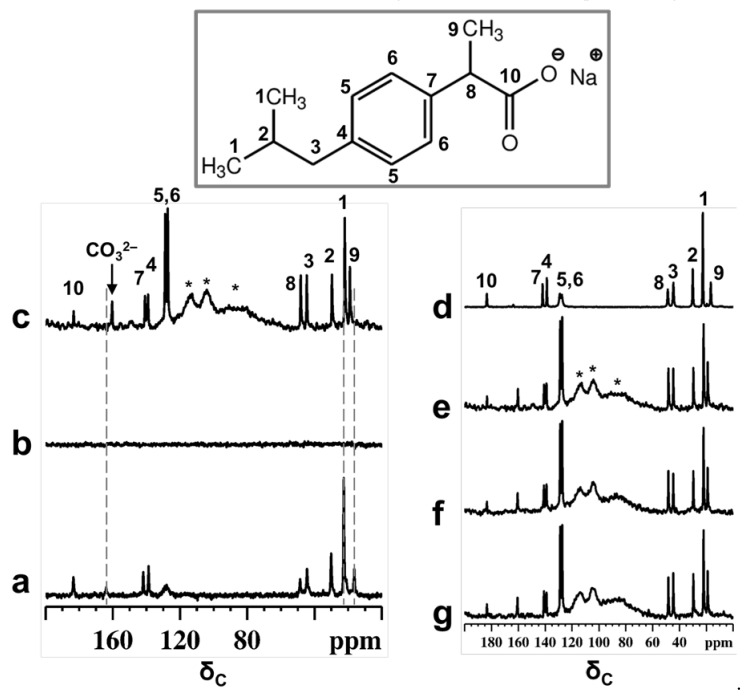
^13^C CPMAS NMR spectra of physical mixture of Ibu-Na and MCM-41 before (**a**) and after contact with D_2_O (**b**). ^13^C MAS NMR spectra of physical mixture after contact with D_2_O (**c**). The NMR spectra are recorded within 30 min of contact with D_2_O. For comparison purpose, the ^13^C CPMAS NMR spectrum of IBU-Na is shown (**d**). Selected patterns of the ^13^C time resolved in situ MAS NMR spectra of physical mixture after contact with D_2_O, time = 30 min (**e**), 420 min (**f**) and 2880 min (**g**). Inset shows the molecular structure of Ibu-Na form with the ^13^C labeling. * indicate peaks due to Kel-F insert and probe background.

^13^C NMR spectra of a physical mixture of Ibu-Na and MCM-41, before and after treatment with D_2_O are reported in Figure 1. The spectral assignments are carried out with the help of liquid-state NMR spectrum (Figure S1) and previous reports [35,36,37,38]. Before contact with D_2_O, the spectra of physical mixture exhibited a typical ^13^C CPMAS NMR spectrum of the Ibu-Na in solid state (Figure 1a). Nevertheless, the peak intensities and chemical shifts indicate that only one peak is present for each ^13^C nuclei. However, upon contact with D_2_O, the ^13^C CPMAS NMR spectrum of physical mixture failed to record signals and confirmed the absence of a residual crystalline solid phase in the mixture. As a matter of fact, a similar experiment on a Ibu-Na/D_2_O (without the presence of MCM-41) mixture resulted in the detection of ^13^C signals in a CPMAS NMR spectrum (Figure S2).

On the other hand, narrow ^13^C signals are displayed in the MAS NMR spectrum (Figure 1c). Besides that, subtle changes in the chemical shift values are noted for selected carbon atoms (Table S3). Previous reports have shown that characteristic changes in the ^13^C chemical shifts values can be considered as a fingerprint of confined ibuprofen located in the mesopores [37,39], especially for carbon C9, which showed a difference of 2.3 ppm. The failure of the CPMAS experiment in the presence of MCM-41 indicates a high degree of mobility for Ibu-Na molecules inside the structure of mesoporous silica and strong reduction in heteronuclear dipolar interactions. Therefore, it can be indisputably stated that Ibu-Na molecules are packed inside MCM-41 mesopores. In addition, a narrow peak at around 161 ppm was detected in the ^13^C spectra and is due to bicarbonate ions originating from the trona and nahcolite (polymorphs of NaHCO_3_) present in the sample. This point will be further demonstrated in the following section.

The adsorption process was monitored in situ for two days; the selected pattern of the time resolved study is reported in Figure 1e–g. No apparent change was observed in the spectra, indicating that the packing of Ibu-Na into MCM-41 is a fast process and was completed in the first minutes of contact. The high degree of mobility for Ibu-Na molecules could arise due to specific motional modes, such as translational, rotational (both isotropic reorientation of the molecules and rotation of isopropyl units or methyl groups) and the flipping of phenyl moieties. Furthermore, the level of the pore filling and their physical state were evaluated. The ^13^C MAS NMR spectra (Figure S3) are capable of revealing the local molecular environments of Ibu-Na in various states, such as in a confined or liquid state. Very subtle variations, such as differences in linewidth and changes in chemical shift values, were noted among the spectra (Table S3). Moreover, two important differences were noted for Ibu-Na molecules trapped inside the MCM-41 mesopores (Figure S3, insets). They are not as highly mobile as in the liquid state (Figure S3 c, insets), as indicated by the narrow linewidth; however, they are not as rigid as in molecules filled by mechanoloading (broader peaks). The mesopores are probably filled jointly by D_2_O and Ibu-Na, leading to their multimodal surface silanol interactions, that reflect the presence of both pore interface and fluidic molecular species [40].

Indeed, a recent study noted that the freedom of motion is restricted in fully packed mesopores compared to partially filled pores [39]. The implication of the ^13^C NMR spectral observations is that dynamic disorder may exist when Ibu-Na adsorbs from water, while static disorder, due to multiple adsorption sites and geometries, may dominate in the mechanoloaded (without the assistance of any solvents) sample [41,42]. While the latter process is predominantly surface docking in nature, leading to a distribution of ^13^C chemical shifts, it can still be partially dynamic, as the mesopore surface can undergo rearrangement in response to the transient molecular adsorption. The former process would allow the faster adsorption of organic molecules on mesopore surfaces, assisted by the shuttling capability of the solvent molecules from the bulk. Nevertheless, the water content, both inside and outside the mesopores are the driving force of the adsorption process in such systems, leading to a rapid averaging of the ^13^C resonance peaks, and may influence the adsorption rate and mobility of the Ibu-Na molecules.

^1^H MAS NMR data provide further insights on the mechanism of molecular adsorption. Prior to the time-resolved in situ ^1^H MAS experiments, the proton spectrum of physical mixture was recorded (Figure S4). The ^1^H MAS NMR spectrum of MCM-41 (middle pattern) exhibited a sharp peak at around 2.27 ppm with a shoulder up-field (lower ppm values) at 1.9 ppm, due to physisorbed water and isolated silanols, respectively. In addition, a very broad peak in the range 2–7 ppm was also clearly visible and is due to hydrogen bonding protons involving silanols and H_2_O. In the ^1^H MAS NMR spectrum of the physical mixture, a peak due to isolated silanols was detected at 1.9 ppm; however, the water peak was down-field shifted to 5 ppm. Furthermore, peaks due to Ibu-Na protons showed no apparent change in their chemical shift values. These NMR data reveal that probably weak interactions between Ibu-Na molecules and MCM-41 surfaces are already taking place in the physical mixture. In addition, low intensity peaks at around 13.8 and 18.3 ppm were detected and are attributed to the presence of nahcolite and trona, respectively (polymorphs of NaHCO_3_) [43].

**Figure 2:**
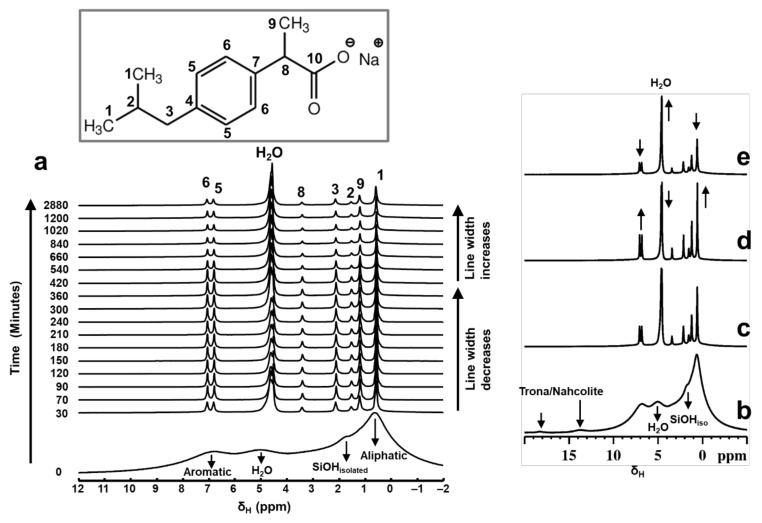
Stacked plot of the time-resolved in situ ^1^H MAS NMR spectra (**a**) showing the rapid adsorption of Ibu-Na on MCM-41 from physical mixture. Each spectrum was collected with 12 s of acquisition time. Selected patterns from the time-resolved in situ ^1^H MAS NMR data showing the variations in peak intensity and linewidths as a function of time; time = 30 min (**c**), 420 min (**d**) and 2880 min (**e**). For comparison purposes, the ^1^H MAS NMR spectrum of physical mixture before contact with D_2_O is shown (**b**). Inset shows the molecular structure of Ibu-Na with ^13^C labelling.

The stacked plot of time-resolved in situ ^1^H MAS NMR data is shown in Figure 2a, recorded up to two days, showing exemplar results for the physisorption behavior of Ibu-Na into MCM-41. A dramatic change in the appearance of spectral pattern is clearly evident when one compares the ^1^H MAS NMR spectra recorded at time = 0 and time = 30 min. Upon initial contact with D_2_O (time = 30 min), the Ibu-Na protons exhibited very sharp resonance peaks, due to the motional averaging of the strong homonuclear ^1^H-^1^H dipolar couplings. In addition, multiple resonances due to water molecules have started to emerge in the 4.4 to 4.8 ppm range. The up-filed shift as well as the multiple δ_H_ values suggests the presence of bulk water in diverse environments [44]. Peaks due to silanols are never detected in the hydrated physical mixture. One possible explanation for their absence is that they are in fast chemical exchange with D_2_O.

Furthermore, the ^1^H resonances arising from the hydrogen bonded protons at 13.8 and 18.3 ppm are not detected in the hydrated physical mixture. As the dissolution of Ibu-Na progressed further (time beyond 30 min), relative intensities of the resonance peaks increased along with further homogeneous narrowing. The observed line widths are in the range between 20 Hz and 80 Hz. A closer examination of the in situ ^1^H NMR spectra (Figure 2d) showed that the maximum line narrowing for Ibu-Na protons is detected at time = 420 min, beyond which further broadening is observed. Interestingly, the narrowest line width for water peaks is also achieved at the same time interval.

**Figure 3:**
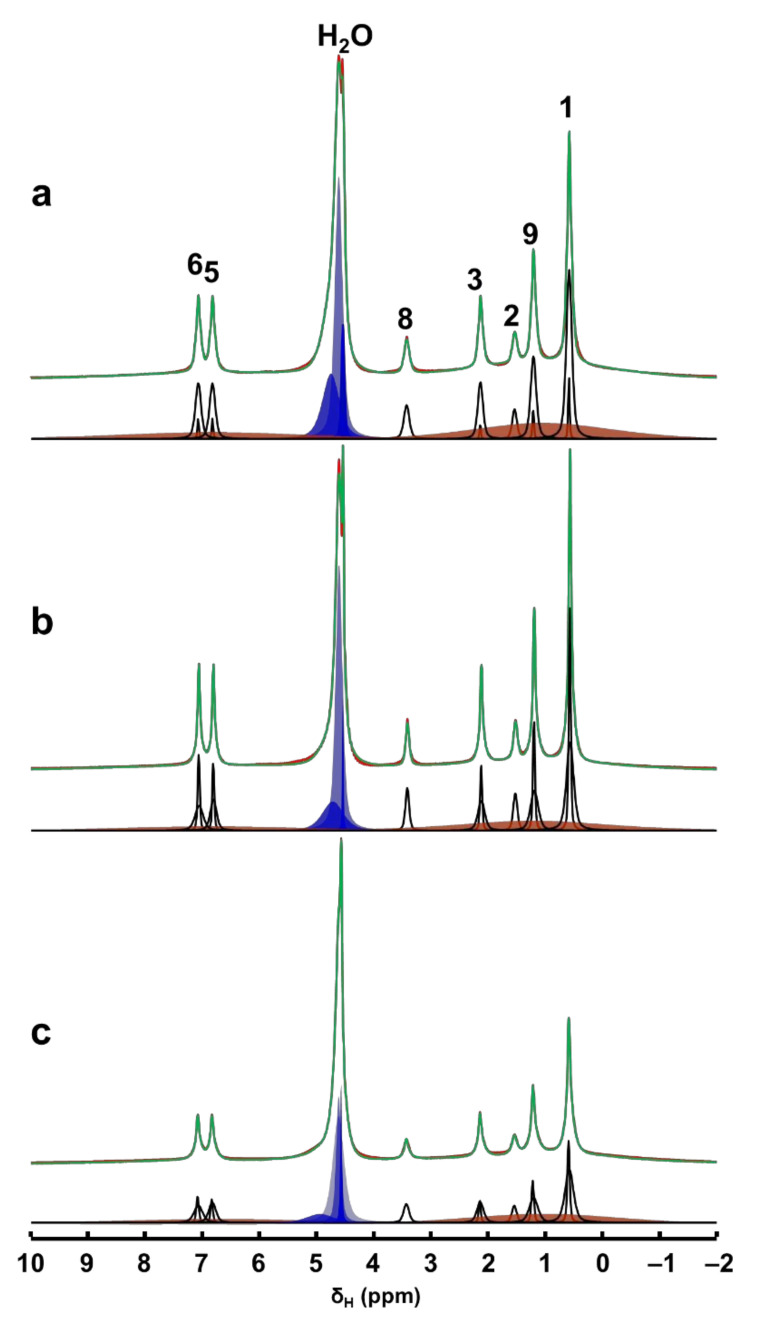
Time-resolved in situ ^1^H MAS NMR spectra, time = 30 min (**a**), 420 min (**b**) and 2880 min (**c**) of the hydrated physical mixture (red) along with the deconvoluted spectrum (green) and the individual contribution from each mobile ^1^H sites (black). The contributions from the solid Ibu-Na are shown in broad orange peaks. Various water environments are shown in blue peaks.

Further quantitative information on the evolution of adsorption process and the progress of mesopore filling in MCM-41 is obtained from the deconvolution of ^1^H MAS NMR spectral data. In Figure 3, we show the deconvoluted ^1^H MAS NMR spectra, which are typical for mixtures of rigid and mobile components with variable mobility, confirming the multimodal distribution of the spectral signals. The undissolved Ibu-Na in the wet physical mixture is represented by broadened aliphatic and aromatic ^1^H resonance peaks (orange peaks). On the other hand, ^1^H MAS NMR spectra are dominated by very sharp resonance lines with a very good resolution for a solid sample, even at moderate MAS frequency of 12 kHz. This interpretation is further supported by a comparison of solid-state and liquid-state ^1^H NMR data of Ibu-Na that reveal the spectral nature of the two states (Figure S5).

Interestingly, at least two different sets of narrow resonances are clearly present for each ^1^H site. The detection of two sets of sharper peaks highlights the presence of two motional regimes within the physisorbed sample. Such sharp lines (line widths between 20 and 80 Hz) are due to motional averaging of the homonuclear ^1^H-^1^H dipolar couplings coming from the reorientation of Ibu-Na in the mesopores of MCM-41. A closer examination of the deconvoluted ^1^H MAS NMR spectra at different time intervals illustrated the diverse intensity ratio between the two sets of sharper peaks. At the beginning of the adsorption process, among the sets of sharper peaks, relatively broader resonances have dominated the spectrum. However, at time = 420 min, narrower resonances emerged as the most intense contributor to the spectrum. Toward the end of experimental study, the trend again reversed, meaning some form of solidification was initiated.

The sharper resonances are due to Ibu-Na molecules encapsulated inside the mesopores, while the remaining fraction of narrow resonances are attributed to the hydrated Ibu-Na molecules at the external surfaces. As the adsorption progressed in time, more Ibu-Na molecules were filled in the pores. However, once the pores are completely filled, solidification of Ibu-Na was initiated. The presence of such distinct molecular ensembles subjected to intrinsically diverse local molecular environments and exhibiting different dynamical behavior was reported in organic molecules embedded in MCM-41 [45,46]. This hypothesis is further supported by the behavior of water molecules, which exhibited diverse chemical environments within the MCM-41. When water is added, it can remain as physical water or fill the mesopores or interact with surface silanol sites [47,48]. Therefore, the diverse chemical shifts displayed in the ^1^H MAS NMR spectra can be attributed to water molecules outside and inside the pore surfaces interacting with Ibu-Na molecules as well as silanols. Nevertheless, the concurrent dynamic exchange between *’in’* and *‘out’* guest molecules (water and Ibu-Na) explain the rapid adsorption kinetics at ambient temperature. The interplay between various guest–guest and guest–host interactions co-present as either London dispersion forces and/or as hydrogen bonds, and are responsible for adsorption and extreme nanoconfinement.

### 3.3. Time-Resolved In Situ Powder X-ray Diffraction

**Figure 4:**
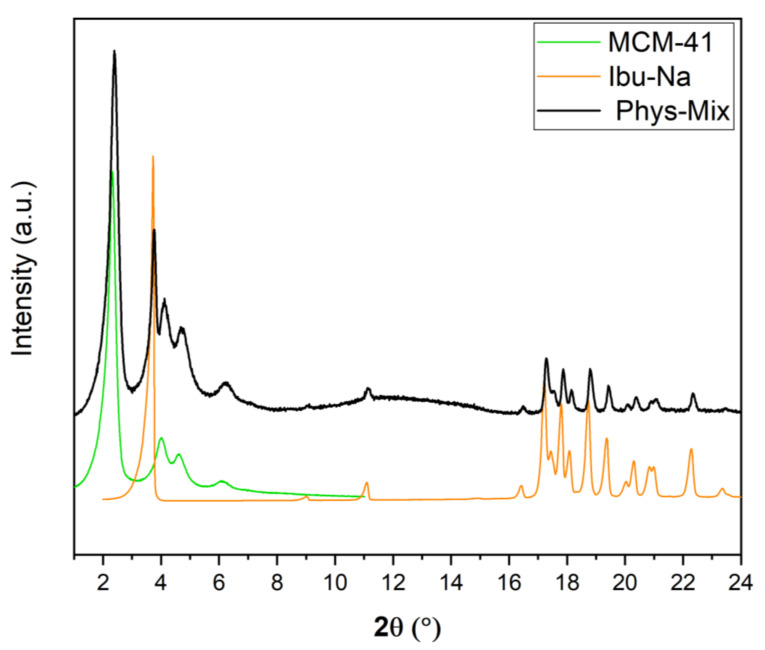
Powder XRD pattern of Ibu-Na, MCM-41 and their physical mixture.

It is also interesting to study the physisorption phenomenon from the crystallographic point of view; for this purpose, time-resolved in situ powder X-ray diffraction studies were carried out. In Figure 4, we show the powder XRD pattern of physical mixture along with MCM-41 and Ibu-Na. The diffraction pattern of pure Ibu-Na displays its high crystalline nature. The main peak at 3.73° 2θ corresponding to (001) Bragg peak is particularly intensive and distinctive [49]. In addition, intense peaks at 11.21°, 17.45°, 17.88°, 18.58°, 19.03°, and 19.77° 2θ are clearly visible. The MCM-41 displayed four typical well-resolved low-angle XRD peaks, which can be indexed into (100), (110), (200) and (210) Bragg peaks reflecting the 2D hexagonal symmetry, indicating the good structural quality of MCM-41 [50]. Moreover, the XRD peaks associated to MCM-41 showed no shift or broadening for the physical mixture. The main phases identified through the Bragg peaks in the physical mixture are sodium salt of Ibuprofen (COD 4510388) and MCM-41. In addition, traces of polymorphs of sodium bicarbonates, such as trona (COD 907656) and nahcolite (COD 1011016) are also detected (Figure S6).

Figure S7 depicts the Ibu-Na structural changes during the contact with water probed by the time-resolved in situ powder XRD technique. Several representative powder XRD patterns, which show the dynamic crystal transformation during the uptake of water, are selected and are presented in order to confirm the formation of the dihydrate state from the anhydrous form. It is reported that the transition between the anhydrous to hydrated state, and vice versa, of Ibu-Na is fast and can be found both as dihydrate and anhydrous racemic compound or anhydrous conglomerate forms [49,51]. On the other hand, it is known that the conversion of racemic anhydrous into hydrated state removes disorder and leads to a more crystalline state [52]. Geppi and co-workers found that the anhydrous form hydrated rapidly and the conversion was almost completed in an hour’s time [49]. As expected, the isostructural hydrated form remained stable over the course of the in situ experimental study (Figure S7). These results are in line with the literature data [49,51].

**Figure 5:**
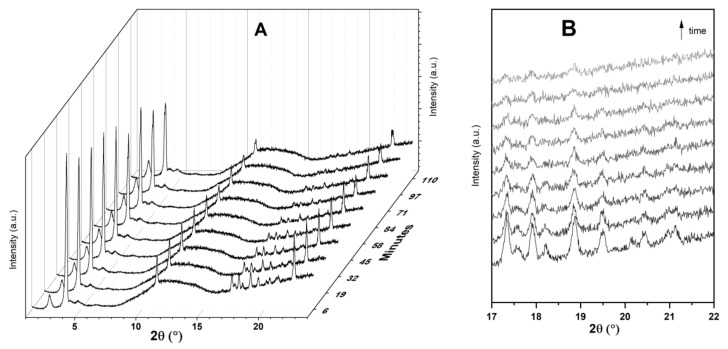
Time resolved in situ powder XRD patterns (**A**) collected during the physisorption of Ibu-Na on MCM-41. The zoom version of the selected range showing the disappearance of diffraction peaks (**B**). The broad peak, between 8° and 16° 2θ is due to background signal from the dome.

Figure 5 displays time-resolved in situ powder XRD patterns collected during the physisorption of Ibu-Na on MCM-41. The powder XRD data revealed that there are two competing processes that occur simultaneously, namely, water uptake to form dihydrate form and the physisorption of Ibu-Na into mesopores of MCM-41. Initially, the former process dominates as the peaks of the anhydrous form at 3.82° 2θ decrease in intensity and dihydrate forms as its reflection at 3.73° 2θ increases. However, at later stages, the diffraction peaks associated to Ibu-Na start to gradually disappear as physisorption dominates (Figure 5B). Thus, the Ibu-Na molecules embedded within the mesopore channels are in a molecularly dispersed state with no long-range order. On the other hand, as the impregnation process started, a significant decrease in the diffraction peak intensity corresponding to the (100) reflection of MCM-41 was noted and is consistent with the mesopore filling with Ibu-Na and water molecules [53]. Overall, the time-resolved in situ powder XRD data suggest that the physisorption of Ibu-Na molecules into the pores of MCM-41 occur rapidly and exist in an amorphous and stable state.

### 3.4. Ibu-Na Adsorption from Synthetic Urine

The adsorption of Ibu-Na from the synthetic urine medium onto selected inorganic adsorbents was performed at pH = 6 (Table 2). Cloisite-based clays do not show any efficiency in Ibu-Na adsorption from synthetic urine medium, and the electrostatic repulsion between clay and Ibu-Na dominates, which mainly present as an anion. It is deduced that in this setting, a drug like Ibu-Na fails to compete effectively for the active sites of the clays. On the other hand, ordered mesoporous MCM-41 and hierarchical ZSM-5 showed significant adsorption capacity.

Ordered mesoporous MCM-41 is confirmed as the best adsorbent among the inorganic matrixes, followed by hierarchical ZSM-5. The binding mechanism mainly proposed for Ibu-Na adsorption on mesoporous silica is the formation of non-covalent interactions with the pore surface. However, when one compares the adsorption of Ibu-Na from water and synthetic urea, it is clear that there is a decrease in the adsorption capacity for the latter system. It is also necessary to keep in mind the complex competition that may exist for active sites in the mesopores between Ibu-Na and the organic components of synthetic urine, such as urea and citrate. Urea is a neutral molecule that can efficiently form hydrogen bonds, and citrate is a triprotic acid with pKa at 3.1, 4.8, 6.4, and could be more competitive. In light of these data, it can be formulated that a significant percentage of PhACs could also be removed from either water or synthetic urine, using adsorbents, such as MCM-41 or hierarchical ZSM-5.

Furthermore, the adsorption of Ibu-Na from synthetic urine medium at pH = 4 and pH = 8 was performed and the data are shown in Tables S4 and S5, respectively. Exceptional adsorption capacities for Ibu-Na are displayed by ordered mesoporous MCM-41 and hierarchical ZSM-5 at a pH value of 4. On the other hand, a significant drop in the efficient adsorption and packing of Ibu-Na in the studied inorganic materials was noted at a pH value of 8.

### 3.5. Raman Spectroscopy

**Figure 6:**
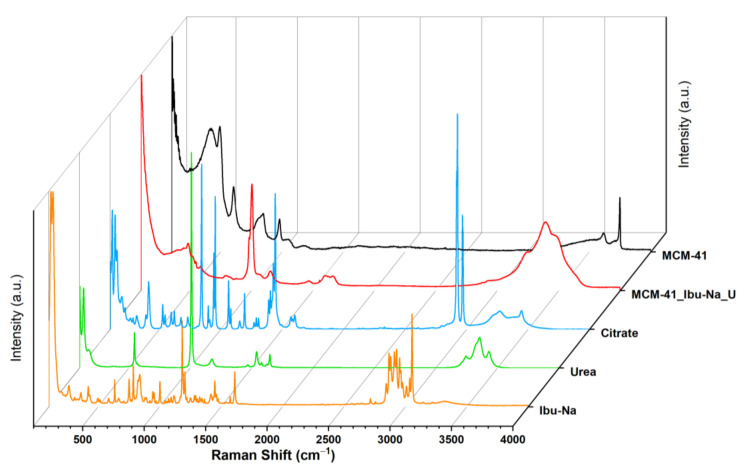
Raman spectra of MCM-41 (black), MCM-41\Ibu-Na_U (red), citrate (blue) urea (green), Ibu-Na (orange).

The uptake process and the ensuing competition were monitored, using Raman spectroscopy. The acronym MCM-41\Ibu-Na_U identifies the sample we measured after the Ibu-Na adsorption test from the synthetic urine medium. The Raman spectrum of MCM-41\Ibu-Na_U sample (Figure 6) appears radically different from that of the corresponding starting materials, such as Ibu-Na, urea, citrate and MCM-41. The disappearance of the signal due to isolated silanols (3750 cm^−1^) and the appearance of bands due to O-H and N-H groups (at 3620, 3478, 3393 and 3232 cm^−1^) are observed [54]. The Raman profile of MCM-41\Ibu-Na_U is very similar to that of urea; similarities are evident in the range 3250–3500 cm^−1^, with three wide bands originating from the N-H stretching of the organic molecule, and more indicatively, the intense peak at 1010 cm^−1^, attributable to the symmetrical stretching of C-N accompanied by a weaker signal at 1150 cm^−1^, its asymmetric stretching appears instead at 1466 cm^−1^. Its contribution to the 1600–1660cm^−1^ band of MCM-41\Ibu-Na_U is also evident: in this window, the stretching motions of the carbonyl (1540 cm^−1^) and of deformation of the NH_2_ groups (1650 and 1620 cm^−1^) of urea fall [55]. In the range between 100 cm^−1^ and 1800 cm^−1^, significant differences between MCM-41 and MCM-41\Ibu-Na U are observed; in the spectrum of the latter, the silica signals are dominated by the appearance of an intense peak centered at 1004 cm^−1^ accompanied by a wide profile at 1100 cm^−1^ and a band clearly distinguishable at 1157 cm^−1^. A complex band is present in the range 1600–1660 cm^−1^, probably originated from the addition of several vibrational motions that fall into the region, and a weaker signal at 1460 cm^−1^, being strongly asymmetrical.

**Figure 7:**
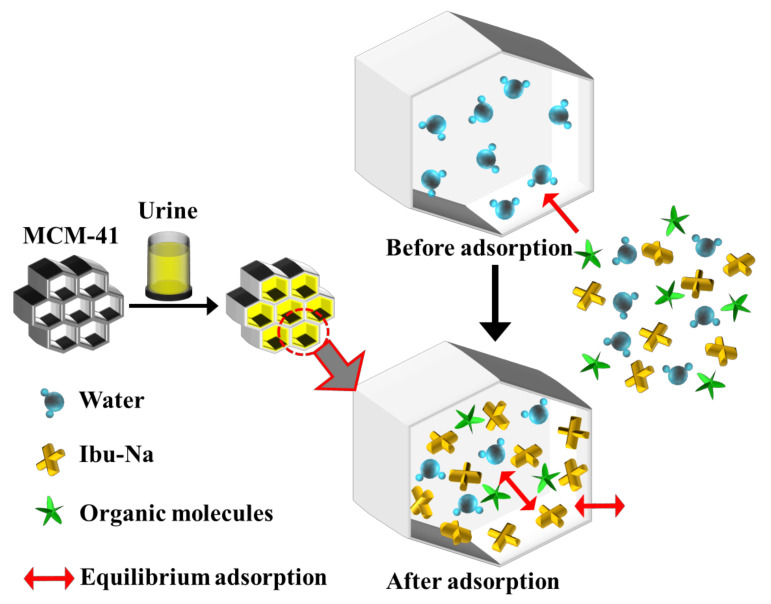
A scheme describing the mechanism of transport and equilibrium adsorption of Ibuprofen from urine.

From the spectra, it is possible to recognize the presence of Ibu-Na and citrate by the signals at 2950 cm^−1^ and stretching region of C-H [56,57]. A slight shift in the peak of urea C-N stretching is also visible (1010 cm^−1^). The region at 1400–1800 cm^−1^ appears very complex; in this range, typical signals due to the stretching of the carbonyls of different organic components of synthetic urine and to the deformation modes of the groups NH_2_ can be assigned. The broadening of signals may indicate their involvement in interactions with silica or other molecules. In conclusion, urea is confirmed as the dominant component in the Raman profile of MCM-41\Ibu-Na_U and is, therefore, supposed to constitute the predominant fraction of organic molecules adsorbed on MCM-41, thus qualifying as the main interfering factor in synthetic urine adsorption. On the other hand, the contribution of citrate is limited. In Figure 7, we show a scheme describing the mechanism of transportation and equilibrium adsorption of Ibuprofen from urine.

## 4. Conclusions

This study has demonstrated that the emerging contaminants, such as drugs and active pharmaceutical ingredients, can be effectively removed from polluted water or urine at their source by adsorption. The optimum adsorbent materials for the easier removal of drugs and metabolites were found to be ordered mesoporous silica (MCM-41), followed by hierarchical zeolites and clays. The uptake process, the ensuing competition, the efficiency and selectivity as well as the packing of model compound, Ibu-Na, in MCM-41 were all thoroughly monitored by a novel approach involving time-resolved in situ ^1^H and ^13^C MAS NMR spectroscopy as well as X-ray powder diffraction.

The time resolved in situ multinuclear MAS NMR spectroscopic study has successfully illustrated the role of MCM-41 as an Ibu-Na carrier and adsorbent. The direct observation of molecularly adsorbed Ibu-Na on the mesoporous surfaces of MCM-41 by MAS NMR revealed the potentials of a newly designed time-resolved in situ NMR approach. Water molecules facilitate the transportation and eventually the adsorption of organic molecules on mesoporous surfaces; however, the cooperative and dynamic interactions between water, silica surface and organic molecules are envisaged. Subsequently, in agreement with the findings in this study, an improvement in the physisorption of active pharmaceutical ingredients (API) can be achieved by employing ordered porous silica or high surface area materials with the aid of solvents.

We have elucidated that by the meticulously designed time-resolved multinuclear MAS NMR spectroscopy, it is possible to follow the adsorption of Ibu-Na in situ, for that matter, any organic compound, to a porous system such as MCM-41 by incipient wetness impregnation. The fundamental mechanistic steps of the encapsulation advancement with time and the transient molecular adsorption as well as the final local molecular state of the adsorbed Ibu-Na molecules in a wet porous sample, that are inaccessible by other means, are established here. Furthermore, the time-resolved in situ X-ray diffraction approach facilitates the monitoring of multi-step molecular adsorption, which complements the NMR data. Finally, this study proposes the use of combined complementary in situ tools to provide unprecedented insights as well as to establish and visualize complex processes, such as molecular adsorption.

## Figures and Tables

**Table 1:** Ibu-Na adsorption capacity of various inorganic adsorbents from physiological saline solution.

Adsorbents	Adsorption (±2)%	Loading (mg/g)
Cloisite-Na	0	0.02
Cloisite-Ca	4	0.16
Sieve (N.A.) ^1^	0	0.02
Sieve (Act.) ^2^	2	0.08
Hier ZSM-5	14	0.74
MCM-41	42	1.94
MCM-41 NPs	45	1.90
Silica gel	4	0.19
Fumed silica	4	0.17

^1^N.A.; not activated. ^2^ Act.; activated.

**Table 2:** Adsorption of Ibu-Na from synthetic urine at pH 6 on selected inorganic adsorbents.

Adsorbents	Adsorption (±2)%	Loading (mg/g)
Cloisite-Na	0	0
Cloisite-Ca	1	0.09
MCM-41	28	1.40
MCM-41 NPs	21	1.03
Hier ZSM-5	26	1.32

## Data Availability

All data contained within the article or supplementary material.

## Supplementary Materials

The following are available online at https://www.mdpi.com/article/10.3390/ma14206196/s1, Table S1: The concentrations and amounts of synthetic urine components used throughout the experiments, Table S2: Parameters used for calculating the initial concentration of active substance, Table S3: ^13^C chemical shift (ppm) assignments of Ibu-Na in liquid state, solid state, confined state with and without water in MCM-41, Table S4. Adsorption of Ibu-Na from synthetic urine at pH 4 on selected inorganic adsorbents. Table S5. Adsorption of Ibu-Na from synthetic urine at pH 8 on selected inorganic adsorbents. Scheme S1: Tools for time-resolved in situ MAS NMR experiments, 4 mm zirconia rotor and Kel-F cap (A), Kel-F insert, plug and screw (B). Tools for time-resolved in situ powder XRD experiments, low background and airtight PMMA specimen holder with a sample reception of 20 mm diameter silicon wafer with cavity (C), a dome like cap equipped with a knife edge beam stop and a one Euro coin, Figure S1: ^13^C CPMAS NMR spectra of ibuprofen in acid-form (a) and sodium salt-form (b) showing the differences in their crystallographic states. For comparison purpose, the liquid-state NMR spectrum of ibuprofen in sodium salt-form collected using D_2_O as solvent is also shown (c). Inset shows the molecular structure of ibuprofen in sodium salt-form with the ^13^C labelling. * indicates peaks due to spinning side-bands, Figure S2: ^13^C MAS (a) and CPMAS (b) NMR spectra of Ibu-Na mixed with D_2_O. The NMR spectra were recorded within 10 min of contact between ibuprofen and D_2_O. For comparison purpose, the ^13^C CPMAS NMR spectrum (c) of Ibu-Na before contact with D_2_O is also shown. * indicates peak due to probe background, Figure S3: ^13^C MAS NMR spectra of physical mixture after 1200 min of contact with D_2_O (a), Ibu-Na loaded in MCM-41 with mechanoloading (without any solvents) (b) and the liquid-state NMR spectrum of Ibu-Na in D_2_O (c). Insets show the zoom spectra. * indicates peak due to either probe background or Kel-F insert, Figure S4: ^1^H MAS NMR spectra of physical mixture (a) together with the spectra of MCM-41 (b) and Ibu-Na (c), Figure S5: ^1^H MAS NMR spectra of ibuprofen in acid-form (a) and sodium salt-form (b). For comparison purpose, the liquid-state NMR spectrum of ibuprofen in sodium salt-form collected using D_2_O as solvent is also shown (c). Traces of NaHCO_3_ polymorphs (Trona and Nahcolite) are present in sample b. * denote solvent peak, Figure S6: Powder XRD pattern of Ibu-Na along with the phase determination and identification using EVA software. Peaks from Ibu-Na—COD 4510388 in orange, minor phases such as Trona—COD 907656 in cyan and Nahcolite—COD 1011016 in green, Figure S7: Stacked plot of the time-resolved in situ powder XRD patterns (A) collected during the sorption of water on Ibu-Na. The zoom version of the selected range showing the identical diffraction patterns (B).
