# Supplementary material for: Adsorption Features of Various Inorganic Materials for the Drug Removal from Water and Synthetic Urine Medium: A Multi-Technique Time-Resolved In Situ Investigation
Source: Materials (Basel). 2021 Oct 19;14(20):6196. doi: 10.3390/ma14206196 (PMC8540798; doi:10.3390/ma14206196)
Supplement: Supplementary file 1 [file materials-14-06196-s001.zip › materials-1409040-supplementary.pdf]

Supporting information for

Adsorption Features of Various Inorganic Materials for the Drug Removal from Water and Synthetic Urine Medium: A Multi-technique Time-Resolved In Situ Investigation

Enrico Boccaleri*, Cristina Marzetti, Giorgio Celoria, Claudio Cassino, Geo Paul*, Ivana Miletto* and Enrica Gianotti

Centre, Dipartimento di Scienze ed Innovazione Tecnologica (DiSIT), Università del Piemonte Orientale, Viale T. Michel 11, 15121 Alessandria, Italy; cristina.marzetti@yahoo.it (CM); giorgio.celoria@uniupo.it (GC); claudio.cassino@uniupo.it (CC); enrica.gianotti@uniupo.it (EG)

* Correspondence: enrico.boccaleri@uniupo.it (EB); geo.paul@uniupo.it (GP); ivana.miletto@uniupo.it (IM)

Table S1. The concentrations and amounts of synthetic urine components used throughout the experiments.

Components	M_W (g mol^{-1})	[C] (mmol L^{-1})	Mass (g)
Urea	60.06	250	15.02
NaCl	58.44	44	2.57
Na_2SO_4	142.04	15	2.13
KCl	74.55	40	2.98
$MgCl_2•6H_2O$	203.3	4	0.81
NaH_2PO_4	119.98	20	2.40
$CaCl_2$	129	4	0.44
$Na_3Citrate•2H_2O$	294.1	2.7	0.79

Table S2. Parameters used for calculating the initial concentration of active substance.

Active substance	[1]DDD (mg/day)	[2]F (%)	[3]V_{urine} (mL)	[4][C] (mg L^{-1})	[4][C] (M)
Ibu-Na	1200	3	1500	23.7	1.15×10^{-4}

[1]DDD (Daily Defined Dose), [2]F is the unchanged excreted drug fraction through the urine, [3]V_{urine} the average daily urine volume, [4][C] is concentration.

Table S3. ^{13}C chemical shift (ppm) assignments of Ibu-Na in liquid state, solid state, confined state with and without water in MCM-41.

Atom	Liquid state	Solid state	Confined state (with H_2O)	Confined state (without H_2O)
C1	21.6	22.6	22	21.9
C2	29.7	30	29.6	29.7
C3	44.2	44.5	44.6	44.8
C4	140.5	138.8	139	138.9
C5	129.4	129.1	128.8	128.7
C6	127.2	127.5	127.3	127.2
C7	140.7	141.9	140.9	140.8
C8	48.2	48.7	48.3	48
C9	18.5	16.6	18.9	18.9

C10	184	183.4	183.3	183.5

Table S4. Adsorption of Ibu-Na from synthetic urine at pH 4 on selected inorganic adsorbents.

**Adsorbents**	**Adsorption (± 2) %**	**loading (mg/g)**
Cloisite-Na	0	0
Cloisite-Ca	2	0.08
MCM-41	60	3.02
MCM-41 NPs	44	2.14
Hier ZSM-5	51	2.54

Table S5. Adsorption of Ibu-Na from synthetic urine at pH 8 on selected inorganic adsorbents.

**Adsorbents**	**Adsorption (± 2) %**	**loading (mg/g)**
Cloisite-Na	0	0
Cloisite-Ca	5	0.26
MCM-41	10	0.48
MCM-41 NPs	10	0.51
Hier ZSM-5	10	0.49

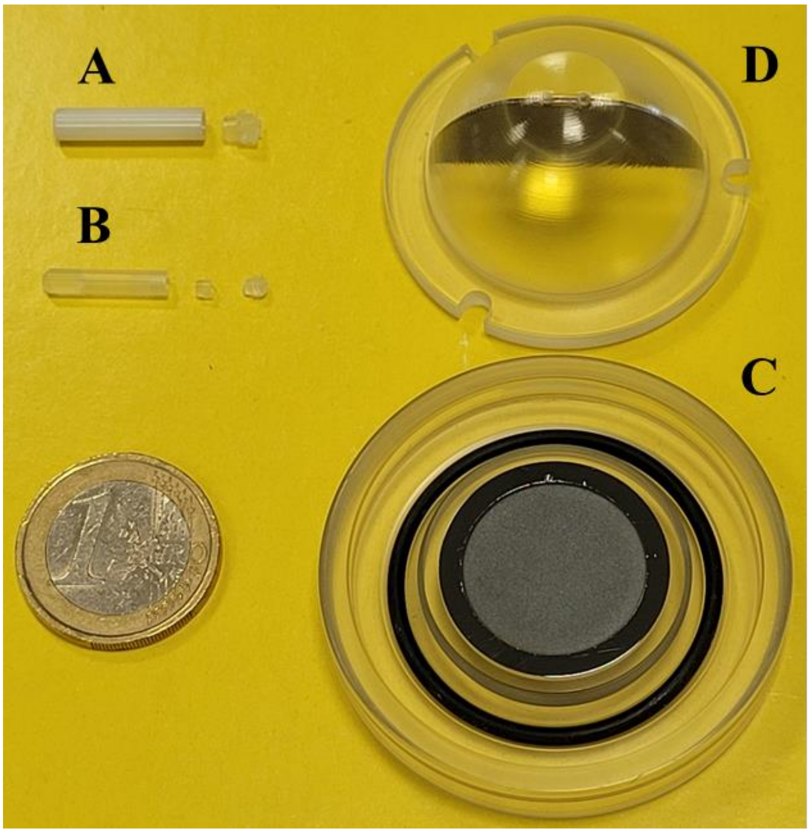

Scheme 1. Tools for time-resolved *in situ* MAS NMR experiments, 4 mm zirconia rotor and Kel-F cap (A), Kel-F insert, plug and screw (B). Tools for time-resolved *in situ* powder XRD experiments, low background and airtight PMMA specimen holder with a sample reception of 20 mm diameter silicon wafer with cavity (C), a dome like cap equipped with a knife edge beam stop (D) and a one Euro coin.

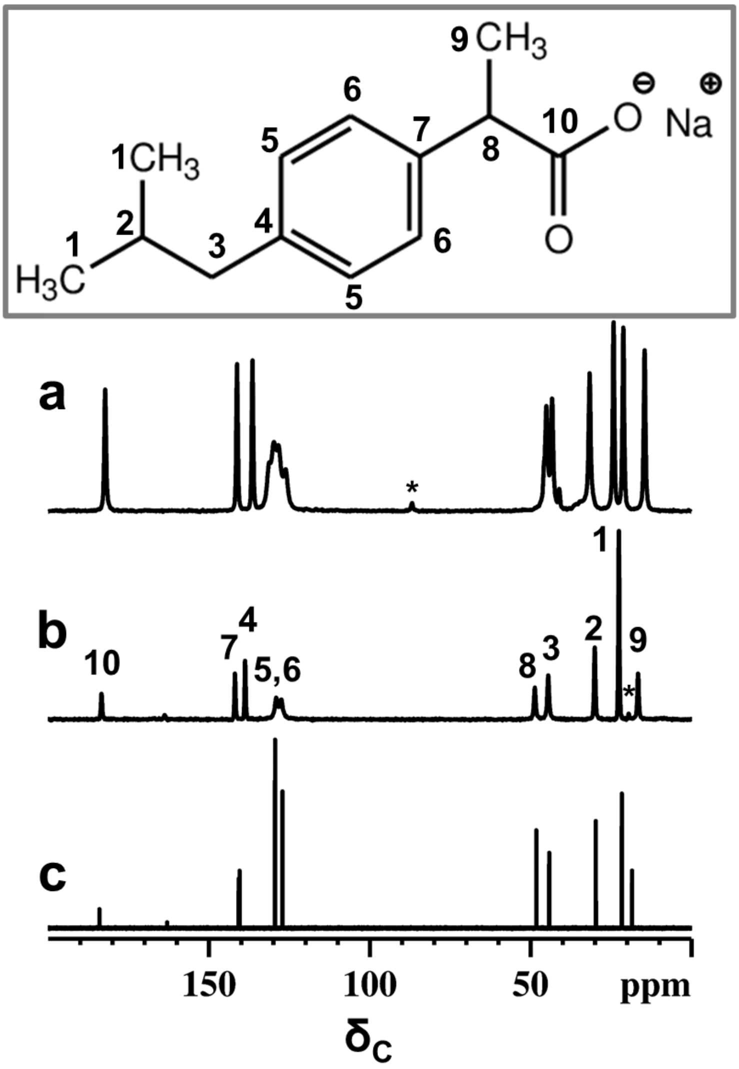

Figure S1. ^{13}C CPMAS NMR spectra of ibuprofen in acid-form (a) and sodium salt-form (b) showing the differences in their crystallographic states. For comparison purpose, the liquid-state NMR spectrum of ibuprofen in sodium salt-form collected using D_2O as solvent is also shown (c). Inset shows the molecular structure of ibuprofen in sodium salt-form with the ^{13}C labelling. * indicates peaks due to spinning side-bands.

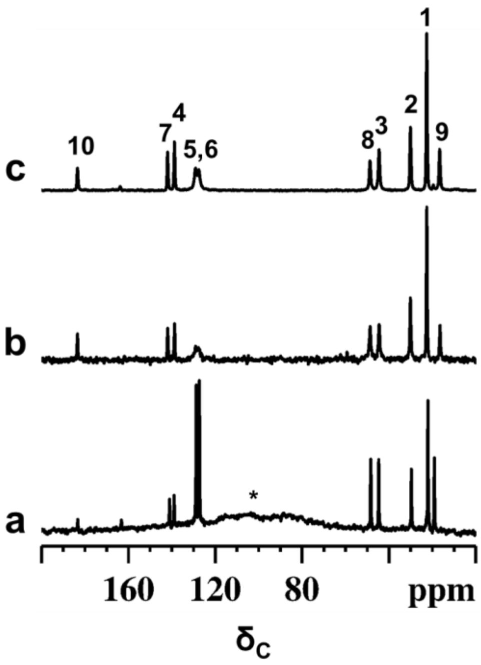

Figure S2. ^{13}C MAS (a) and CPMAS (b) NMR spectra of Ibu-Na mixed with D_2O. The NMR spectra were recorded within 10 minutes of contact between ibuprofen and D_2O. For comparison purpose, the ^{13}C CPMAS NMR spectrum (c) of Ibu-Na before contact with D_2O is also shown. * indicates peak due to probe background.

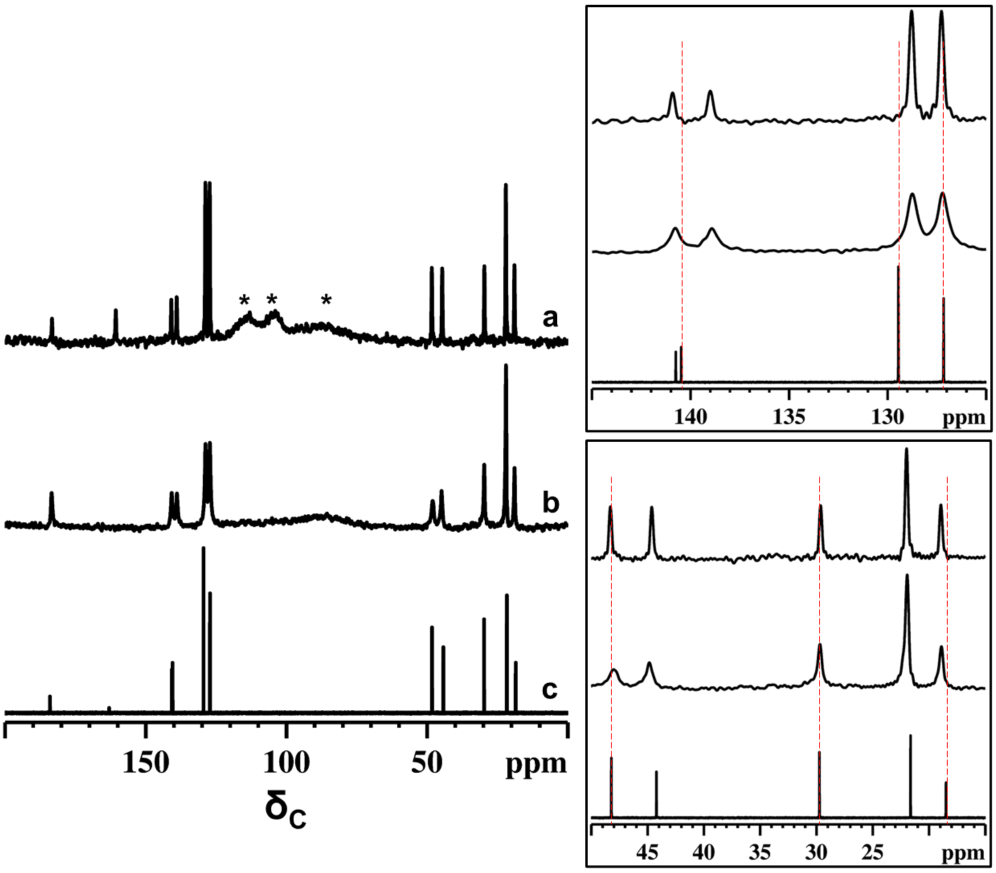

Figure S3. ^{13}C MAS NMR spectra of physical mixture of Ibu-Na and MCM-41 after 1200 minutes of contact with D_2O (a), Ibu-Na loaded in MCM-41 with mechanoloading (without any solvents) (b) and the liquid-state NMR spectrum of Ibu-Na in D_2O (c). Insets show the zoom spectra. * indicates peak due to either probe background or Kel-F insert.

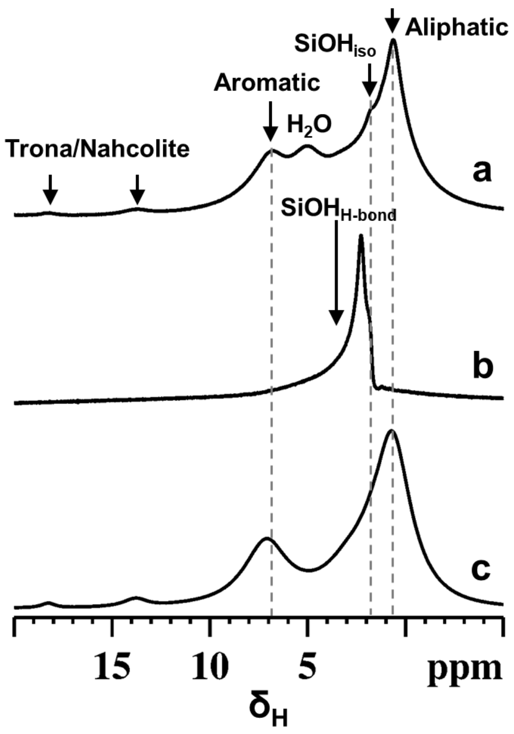

Figure S4. ^{1}H MAS NMR spectra of physical mixture (a) together with the spectra of MCM-41 (b) and Ibu-Na (c).

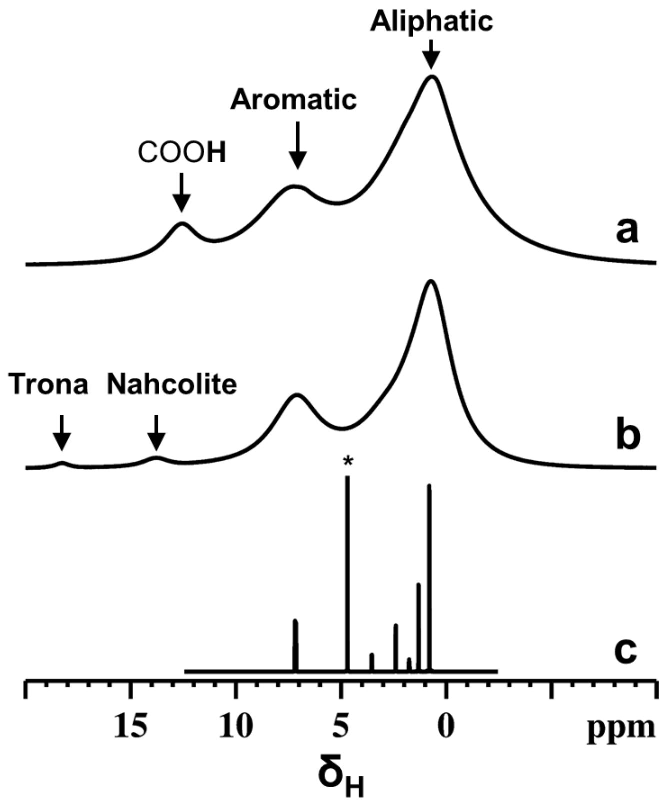

Figure S5. ^{1}H MAS NMR spectra of ibuprofen in acid-form (a) and sodium salt-form (b). For comparison purpose, the liquid-state NMR spectrum of ibuprofen in sodium salt-form collected using D_2O as solvent is also shown (c). Traces of $NaHCO_3$ polymorphs (Trona and Nahcolite) are present in sample b. * denote solvent peak.

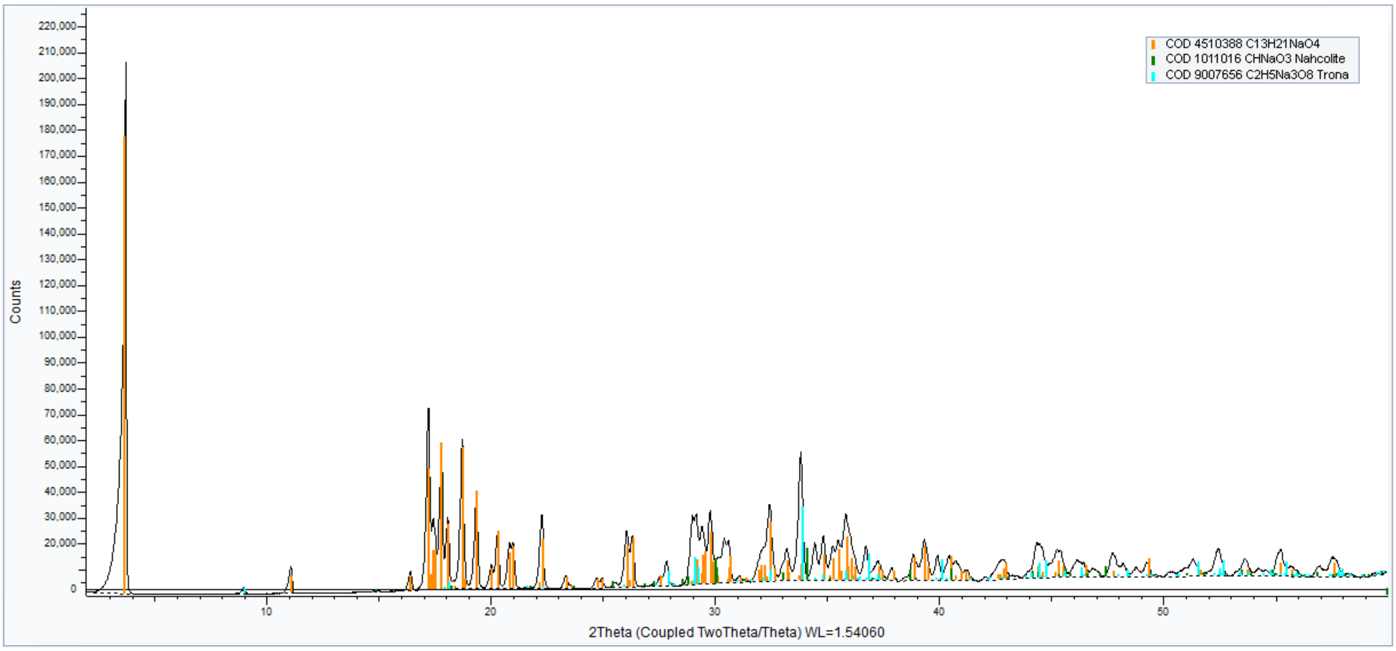

Figure S6. Powder XRD pattern of Ibu-Na along with the phase determination and identification using EVA software. Peaks from Ibu-Na - COD 4510388 in orange, minor phases such as Trona - COD 907656 in cyan and Nahcolite - COD 1011016 in green.

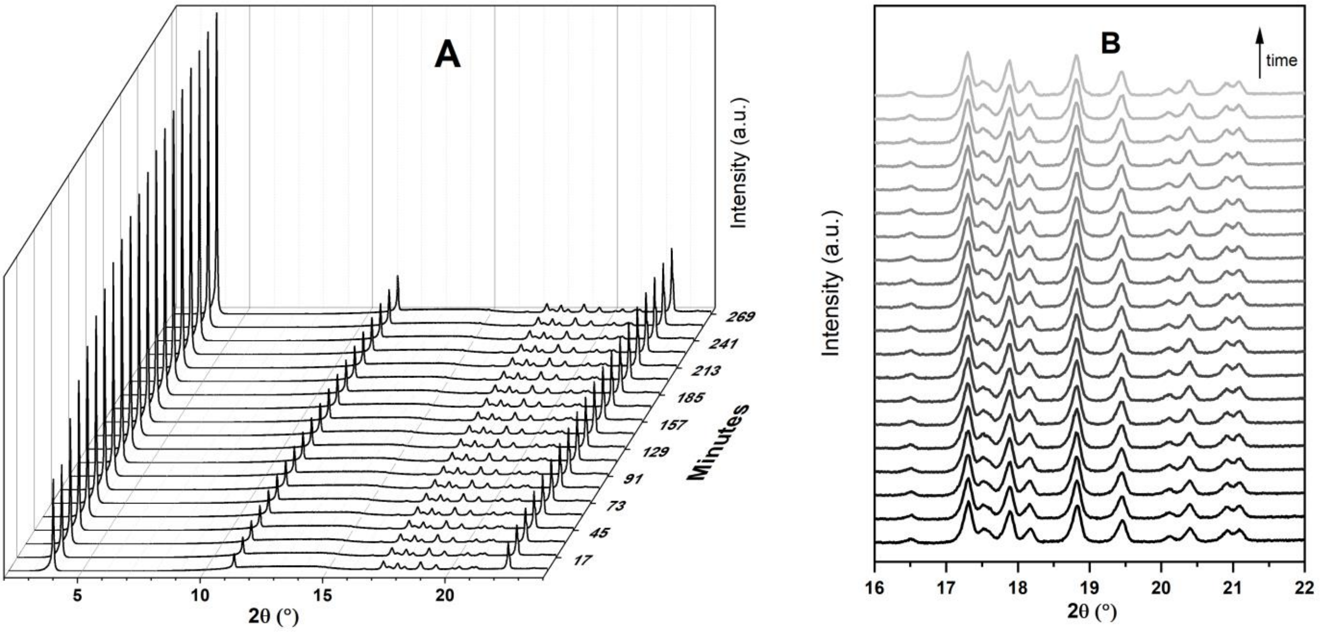

Figure S7. Stacked plot of the time resolved *in situ* PXRD patterns (A) collected during the sorption of water on Ibu-Na. The zoom version of the selected range showing the identical diffraction patterns (B).
